# The Utilization of Lumbar MRI for Lower Back Pain at National Guard Hospital, Jeddah: A Retrospective Cohort Study

**DOI:** 10.7759/cureus.25468

**Published:** 2022-05-30

**Authors:** Emad M Babateen, Ziyad M Alharbi, Waleed K Alnejadi, Mahmoud A Fallatah, Omar R Bukhari, Ahmed Lary

**Affiliations:** 1 College of Medicine, King Saud Bin Abdulaziz University for Health Sciences, Jeddah, SAU; 2 College of Medicine, King Saud bin Abdulaziz University for Health Sciences, Jeddah, SAU; 3 Radiology, National Guard Hospital, Jeddah, SAU; 4 Neurosurgical Oncology, National Guard Hospital, Jeddah, SAU

**Keywords:** guidelines, magnetic resonance imaging (mri), jeddah, lower back pain, management

## Abstract

Introduction

Magnetic reasoning imaging (MRI) is the imaging modality of choice for detecting spinal pathologies. The study of the appropriateness of MRI utilization in Saudi Arabia is lacking. As a result, this research aims to assess the use and misuse of lumbar MRI in lower back pain (LBP) at the National Guard Hospital (NGH) in Jeddah city.

Methods

This is a retrospective cohort study that included all adult patients who had lumbar MRI for LBP at NGH in 2019. A total of 1,225 patients were included. Patients with extreme ages, trauma, recent lumbar spine surgery, spine or spinal canal tumors, and infection were excluded, leaving a number of 805 patients. Specific MRI findings were obtained and assessed in association with history and physical examination.

Results

LBP with radiculopathy was the most common complaint (82.9%) followed by LBP without radiculopathy (12.8%), with the lowest being limb pain alone (2.6%). Overall, 72% of patients had negative MRI findings, which did not explain their symptoms, and 28% had positive MRI findings that were not associated with their symptoms (p < 0.001). A complete physical examination was performed on 27.5% of patients, of which only 12% had positive findings. MRI was ordered for 72.5% of patients without a complete physical examination. Finally, 88.2% of patients who had MRI were managed conservatively, while only 6.7% were managed with surgery (p < 0.04).

Conclusion

The number of patients who had proper assessment prior to the ordering of MRI was significantly low. The decision to request MRI was not based on any scientific basis. This study has demonstrated that without proper and strict guidelines, MRIs will continue to be overutilized, which, in turn, will have negative consequences on the waiting time for an MRI and the cost of all the unnecessary MRIs. Furthermore, a good number of patients nowadays who do not have any indications for an MRI keep asking their physicians for it, and if the physician refuses, they transfer to another physician who will order the MRI.

## Introduction

Lower back pain (LBP) or lumbar pain is the most prevalent condition after hypertension, diabetes, and routine examinations in primary clinics [1]. It has a lifetime prevalence of 39% to 84%, with a peak incidence between 40 and 69 years of age and a slight female predominance [2]. Furthermore, a specific cause cannot be given in 90% of cases, which is called "non-specific LBP” [3]. Also, it is categorized into acute (lasting four weeks), subacute (extending to three months), and chronic (lasting more than three months) [4]. Although it has a recurrence rate of 24% to 80% in a year [5], around 50% to 75% of patients recover spontaneously at four weeks and more than 90% at six months [6]. LBP is ranked as the first cause of activity limitation in young adults [7]. It has a financial burden as the cost of healthcare is increasing rapidly [1].

MRI is the imaging modality of choice for detecting spine pathologies [8]. According to multiple studies, 26% to 44% of lumbar MRIs do not follow the guidelines, which recommend conservative therapy as the new onset LBP approach without red flags [9]. The consequences of ordering unnecessary MRIs do not improve the outcome and have a limited impact on clinical decision-making, as the correlation between image findings and patients' symptoms is not always strong [10]. Additionally, it is associated with an estimated cost of 300 million dollars yearly in the United States [11]. Labeling patients with specific diseases is another catastrophic event associated with imaging, which can affect them permanently [7]. Although the incidence of serious spine pathologies is rare, with an estimation of less than 1%, early imaging does not improve the outcome, and despite the existence of guideline recommendations for more than three decades, still some patients and physicians believe it is useful for non-specific LBP [12-14].

Anxious patients looking for a definitive diagnosis are a major cause of the inappropriate use of imaging [15]. In a survey, 50% of patients believed that everyone with LBP should have imaging, and 72% considered imaging as important [16]. In another survey, it has been found that with patients' insistence, more than one-third of physicians would order lumbar MRI for uncomplicated LBP [17]. On the other hand, some physicians request imaging to take defensive action against sues [18]. Others expect that time to explain and educate patients would take more than only accepting the patient's request to order [19]. Interestingly, a study has shown that 23.6% of physicians were responsible for 74% of overall inappropriate MRI orders [20]. Although this topic was extensively studied in different parts of the world, such results may not be applied to our community due to cultural, environmental, and individual differences. As a result, this research aimed to assess and contribute to the medical literature on the use and misuse of lumbar MRI in uncomplicated LBP at National Guard Hospital (NGH), a non-pecuniary military hospital in Jeddah city, Kingdom of Saudi Arabia.

## Materials and methods

This is a retrospective cohort study that included all adult patients who had lumbar MRI for LBP at NGH in 2019. A total of 1,225 patients were analyzed. Patients with extreme ages (less than 18 years and more than 80 years), trauma, recent lumbar spine surgery, spine/spinal canal tumors, and infection were excluded, leaving a number of 805 patients. The data collected in this research was self-administered. In order to collect patients’ data, we received consent from National Guard Health Affairs in Jeddah to access the documents and electronic records of the selected patients, and it was accessed only by the investigators. The data of interest were collected by a data sheet from the medical records department through the Best Care system, and the data were documented in an Excel sheet. This study was approved by the hospital’s Institutional Review Board.

Main variables

The first part consisted of demographical information that had the following variables: age, gender, and body mass index (BMI). The second part consisted of clinical data covering: symptoms, examinations, and red flags. Also, MRI results included spondylosis, spondylolisthesis, spinal stenosis, neural foraminal stenosis, annular tear, and facet arthropathy. The third part had information regarding the type of action based on clinical and MRI findings: conservative management or surgery. Furthermore, physician specialty ordering the MRI included orthopedics, neurosurgery, neurology, family medicine, pain management, and internal medicine. The MRI findings were reviewed by two neuroradiologists, and they were assessed in association with history and physical examination.

Data analysis

Data were analyzed statistically using the Statistical Package for the Social Sciences (SPSS) program Version 26 (IBM Corp., Armonk, NY, USA). To assess the relationship between variables, qualitative data were expressed as numbers and percentages, and the chi-square test (χ^2^) was used. Quantitative data were expressed as mean and standard deviation (mean ± SD), and the Kruskal-Wallis test was applied to assess the relationship between the non-parametric variables. A p-value of 0.05 was considered statistically significant.

## Results

The mean age of studied patients was 53.04 ± 14.49 years, 56.1% were females, 82% had chronic back pain before undergoing conservative management (>three months), and only 1.4% had red flags. Most patients (43.5%) were obese. Regarding the symptoms, 57.9% had unilateral radiculopathy, while 24.8% had bilateral radiculopathy. LBP without radiculopathy was found in 13.2%, and lower limb pain (LLP) without back pain was found in only 2.6%. The majority of patients (72.5%) had incomplete physical examination prior to ordering the MRI. With regard to the MRI results, 40.7% had normal findings or mild bulges without compression or narrowing. The duration between ordering the MRI and performing it was more than three months (35.3%). Around 75% underwent an X-ray before the MRI, and 34% had duplicate MRIs, with a mean number of MRIs of 1.47 ± 0.85 times (Table 1).

**Table 1:** Distribution of studied patients according to age, gender, and clinical and radiological data SD, standard deviation; BMI, body mass index; LBP, low back pain; LLP, lower limb pain; CT, computed tomography; MRI, magnetic resonance imaging

Variable	No. (%) or N ± SD
Age (years)	53.04 ± 14.49
Gender	
Female	452 (56.1)
Male	353 (43.9)
Red flag	
No	794 (98.6)
Yes	11 (1.4)
BMI	
Underweight	9 (1.1)
Normal weight	113 (14)
Overweight	270 (33.5)
Obese	350 (43.5)
Severe obese	63 (7.8)
Duration of back pain before the management	
Chronic (>3 months)	660 (82%)
Acute (<3 months)	145 (18%)
Symptoms	
LBP only	106 (13.2)
LBP with unilateral radiculopathy	466 (57.9)
LBP with bilateral radiculopathy	200 (24.8)
LLP without LBP	21 (2.6)
Others	12 (1.5)
Examination	
Incomplete	584 (72.5)
Complete with normal findings	124 (15.4)
Complete with positive findings	97 (12)
MRI results	
Normal or mild bulge without compression or narrowing	328 (40.7)
Bulge with compression and narrowing/ and disc prolapse	477 (59.3)
Correlation with clinical history	
No	322 (40)
Yes	483 (60)
Other images	
No	150 (18.6)
X-ray	607 (75.4)
CT	11 (1.4)
Both	37 (4.6)
Duplicate MRI	
No	531 (66)
Yes	274 (34)
Number of MRIs	1.47 ± 0.85
Duration between ordering and doing the MRI	
<1 month	246 (30.6)
1 months	71 (8.8)
2 months	90 (11.2)
3 months	114 (14.2)
>3 months	284 (35.3)

**Figure 1:**
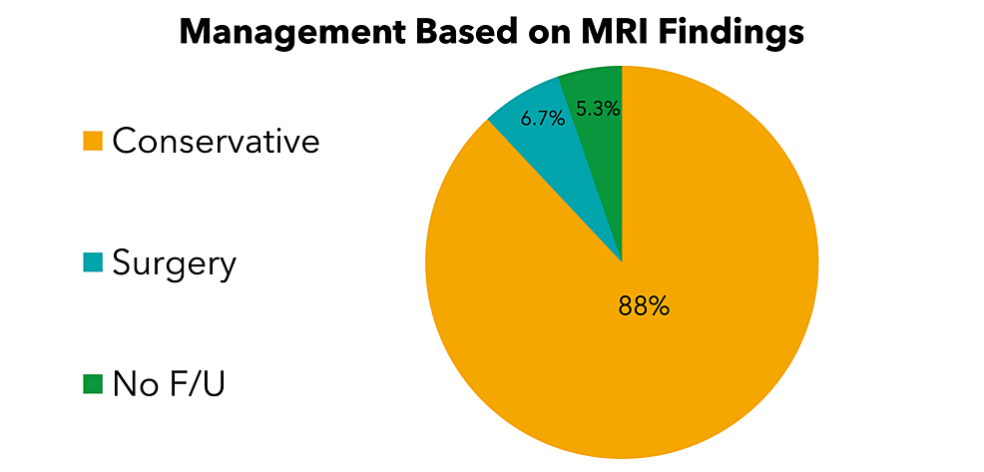
Management Based on MRI findings F/U, follow-ups; MRI, magnetic resonance imaging

Around 88% of patients had conservative management based on MRI findings, as shown in Figure 1.

**Figure 2:**
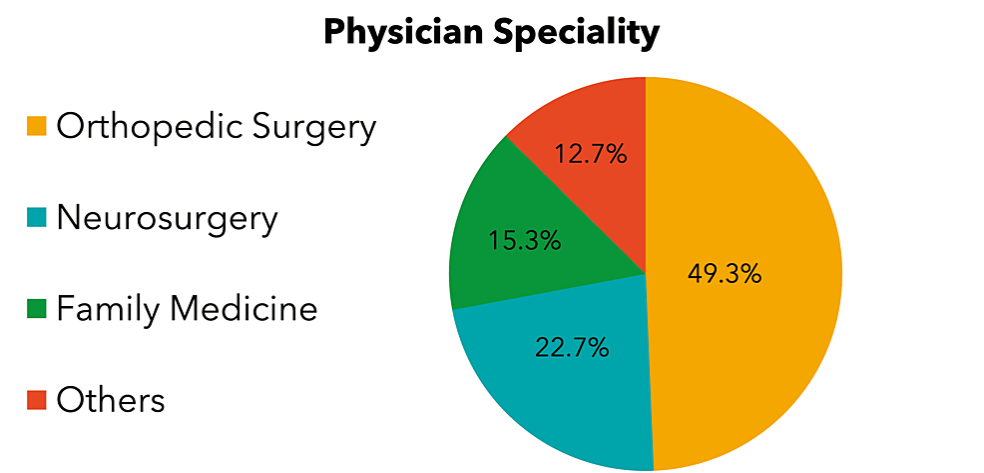
Physician speciality that ordered the MRI MRI, magnetic resonance imaging

Depending on the clinic setting, the number of scans ordered varied greatly with the greatest percentage of MRIs ordered by orthopedics (49.3%), as demonstrated in Figure 2.

The majority of patients who had normal MRI or incidental bulge without compression or narrowing did not undergo proper physical examination (71%). Overall, 71% of patients had negative MRI findings, which did not explain their symptoms, and 19% had positive MRI findings that were not associated with their symptoms (p < 0.001). Also, 26% of those who had duplicated MRI were found to have normal imaging or incidental disc bulges, and 57% of patients who complained of LBP with unilateral radiculopathy had normal MRI or incidental bulge without compression. Most patients who had positive MRI findings underwent conservative management (87.6%), which means that the MRI did not change the management plan. Table 2 summarizes the relationship between MRI results and clinical data.

**Table 2:** Relationship between MRI results and patients’ clinical and radiological data LBP, low back pain; LLP, lower limb pain; MRI, magnetic resonance imaging

Variable	MRI results		χ^2^	p-Value
	Normal or mild bulge without compression or narrowing	Abnormal (bulge with compression and narrowing or disc prolapse)		
Red flags				
No	324 (98.8)	470 (98.5)	0.08	0.766
Yes	4 (1.2)	7 (1.5)	0.08	0.766
Symptoms				
LBP only	48 (14.6)	58 (12.2)	1.96	0.742
LBP with unilateral radiculopathy	188 (57.3)	278 (58.3)	1.96	0.742
LBP with bilateral radiculopathy	79 (24.1)	121 (25.4)	1.96	0.742
LLP without LBP	7 (2.1)	14 (2.9)	1.96	0.742
Others	6 (1.8)	6 (1.3)	1.96	0.742
Examination				
Incomplete	232 (70.7)	232 (73.8)	2.21	0.33
Complete with normal findings	58 (17.7)	66 (13.8)	2.21	0.33
Complete with positive findings	38 (11.6)	59 (12.4)	2.21	0.33
Association with clinical history				
No	222 (70.7)	90 (18.9)	27.82	<0.001
Yes	96 (29.3)	387 (81.1)	27.82	<0.001
Duplicate MRI				
No	244 (74.4)	287 (60.2)	17.51	<0.001
Yes	84 (25.6)	190 (39.8)	17.51	<0.001
Number of MRI	1.36 ± 0.79	1.58 ± 0.88	4.24	<0.001
Management based on findings				
Conservative	292 (89)	418 (87.6)	5.08	0.666
Surgery	17(5.2)	37 (7.7)	5.08	0.666
No follow-ups	19 (5.8)	22 (4.6)	5.08	0.666

A significant number of patients (75.8%) who did not have imaging association with clinical history did not undergo a proper physical examination. Only 12.2% of patients who had imaging association with clinical history had undergone complete examination with positive findings. The majority of physicians who ordered MRI did not complete the patients’ examination, and the most common specialty was orthopedic, followed by neurosurgery (p < 0.012), as shown in Table 3.

**Table 3:** The relationship between physical examination and imaging association with clinical history and physician specialty

Variable	Examination			χ^2^	p-Value
	Incomplete	Complete with normal findings	Complete with positive findings		
Association with clinical history					
No	244 (75.8)	40 (12.4)	38 (11.8)	3.89	0.143
Yes	340 (70.4)	84 (17.4)	59 (12.2)		
Physician specialty					
Family medicine	95 (77.2)	8 (6.5)	20 (16.3)	25.73	0.012
Internist	22 (84.6)	3 (11.5)	1 (3.8)		
Neurosurgery	125 (68.3)	39 (21.3)	19 (10.4)		
Orthopedics	286 (72)	60 (15.1)	51 (12.8)		
Neurology	29 (76.3)	4 (10.5)	5 (13.2)		
Pain management	22 (66.7)	10 (30.3)	1 (3)		
NA	5 (100)	0 (0.0)	0 (0.0)		

## Discussion

In our study, we found that 41% had normal findings in MRI or mild bulges without compression or narrowing. As compared to another study, 79% of the bulging disc or degenerative disc diseases were asymptomatic and commonly seen in MRI but not necessarily to be a source of pain [21]. Furthermore, Emery et al. found that 28.5% were inappropriate MRI findings, and 27.2% were of uncertain value [9]. Also, in our study, we found that 82.7% complained of radiculopathy, which is considered to be self-limiting and will resolve over weeks to months, according to Wáng et al. [22]. Hakelius reported 38 cases of radiculopathy, of which 88% resolved within six months [23]. Furthermore, disc herniation in our study was found in 31.2%, which generally resolves by eight weeks from the onset of symptoms [24]. Moreover, we found that 17% of patients had annular tears, which do not necessarily cause back pain [23]. Brinjikji et al. found that disc annular fissure had no association with back pain [25].

In addition, history and physical examination findings associated with serious diseases are considered to be red flags, which showed an increase in the referral for imaging even though its low specificity is an indication for doing MRI [23]. In our study, we found that only 1.4% presented with red flags, and Henschke et al. found 80% with at least one red flag [12]. One of the potential disadvantages associated with the overuse of lumbar spine MRI in patients with LBP is direct and downstream costs, affecting the future of the healthcare system and patients [21]. In our region, it costs the government yearly 800-1200 Saudi Arabian Riyals, and $300 million dollars per year in the healthcare system in the United States [26].

In our study, 34% had duplicate MRI, with a mean number of 1.47 ± 0.85 times. Of those who had to duplicate MRI, 26 were found to have normal imaging or incidental disc bulges. Compared to another study, Carragee et al. stated that 51 patients had 67 MRI scans, and 43 (84%) had either unchanged MRI or showed regression of baseline changes [27]. Unfortunately, in our study, 584 (72.5%) had incomplete clinical examinations. As stated by Carragee et al., a thorough physical examination including range of motion, the presence of any deformity or tenderness of the thoracolumbar spine, lower extremity neurological examination, and sciatic and femoral root tension signs can often decrease the use of unnecessary MRI and enhance the management outcome [27]. Unfortunately, most patients who did not have MRI findings that explain their symptoms were those who did not have a complete examination (41.7%).

In our study, most patients who had positive MRI findings underwent conservative management (88%), which means that the MRI did not change the management plan. According to Kanaan et al., multiple randomized clinical trials have shown that early imaging versus conservative treatment without imaging for patients with no red flags does not enhance patient outcomes [15]. Moreover, a 2020 study of 405,965 United States primary care patients found that those who had early MRI were more likely to undergo back surgery (1.48% versus 0.12%) and take prescription opioids (35.1% versus 28.6%), yet they had a higher pain score at one-year follow-up (3.99 versus 3.87) than those who did not get early MRI [21]. In our study, 284 (35.3%) patients had to wait for more than three months for an MRI. One study conducted in Canada demonstrated the waiting time issue. Most provinces had wait times between four and six months [28].

Limitations

To evaluate the appropriateness of lumbar MRI, we used the documented data from the hospital system. The system data did not contain the full details of care. Most of the data were extracted based on the physician documentation, which might lack a lot of significant information. Moreover, this study has limited generalizability since it was conducted in one hospital and included only the year 2019 as a sample of assessment. Despite our limited generalizability outside of NGH, analyzing this population provides important insight by demonstrating that even in a system with a large-scale absence of financial and other incentives for overuse, inappropriate ordering of MRIs still remains a significant problem. There should be further investigation into the proportion of inappropriate lumbar MRIs in the Kingdom of Saudi Arabia to have well-organized evidence-based results that would highlight this issue and enhance the healthcare system.

Recommendation

The increased order of unjustified lumbar MRI for LBP is primarily attributable to a lack of established national or institutional guidelines or not following existing guidelines. Globally, several guidelines have been established to avoid ordering inappropriate lumbar MRI in the workup of LBP. All are in the agreement with withholding the MRI in the absence of red flags [29]. These red flags include, but are not limited to, severe or progressive neurologic deficits (e.g., cauda equina syndrome), fever, sudden back pain with spinal tenderness in the background of steroid use or trauma, and serious underlying medical conditions (e.g., cancer, infection). In addition, a useful diagnostic triage tool has been developed by Traeger et al., in which they categorize patients presenting with symptoms of LBP into three broad categories based on focused history and physical examination, and after exclusion of non-spinal causes of LBP [30]. The first category constitutes those with red flags that have specific spinal pathology, in which immediate spinal MRI is indicated. The second and third categories constitute radicular syndrome and non-specific LBP, respectively. Those who do not need MRI should be reassured and managed by alternative pathways such as physical therapy and pain management programs [30].

One more useful and complementary way is to implement a catch-up step in the process of ordering a lumbar MRI; this is especially important for our institution to prevent unjustifiable orders of lumbar MRI. This could be achieved by adjusting the order form of lumbar MRI to include a complete history, physical examination, presence of red flag and its type, and if there were trials of alternatives such as physical therapy and pain management, then the ordering physician must fill them before proceeding with the order. Moreover, this would be optimal with the active involvement of the radiological department as they have a valuable role in identifying and canceling unjustified orders. Therefore, we encourage tailoring national or institutional evidence-based protocol along with a strong system that ensures the recommendations are applied and followed by the physician and their patients.

Patient education is one of the key elements toward better use of lumbar MRI for LBP. Indeed, correcting patients’ misconceptions and negative beliefs about LBP may facilitate the reduction of inappropriate lumbar MRI. We recommend providing patients with information about their condition and emphasizing the favorable prognosis of most LBP.

## Conclusions

The number of patients who had proper assessment prior to the ordering of MRI was low. Most patients who had negative or non-significant MRI findings had an incomplete examination. Ordering MRI did not change the management plan for the majority of patients since most of them were treated conservatively. The decision to request MRI was not based on any scientific basis, which affects the effectiveness of ordering an MRI negatively, and can cause more harm than benefit.
